# Carcinogenesis as Side Effects of Iron and Oxygen Utilization: From the Unveiled Truth toward Ultimate Bioengineering

**DOI:** 10.3390/cancers12113320

**Published:** 2020-11-10

**Authors:** Shinya Toyokuni, Yingyi Kong, Zhen Cheng, Kotaro Sato, Shotaro Hayashi, Fumiya Ito, Li Jiang, Izumi Yanatori, Yasumasa Okazaki, Shinya Akatsuka

**Affiliations:** 1 Department of Pathology and Biological Responses, Nagoya University Graduate School of Medicine, 65 Tsurumai-cho, Showa-Ku, Nagoya 466-8550, Japan; kongyingyi@med.nagoya-u.ac.jp (Y.K.); teishinn@med.nagoya-u.ac.jp (Z.C.); kotarosato1123@med.nagoya-u.ac.jp (K.S.); obgy86str3005@med.nagoya-u.ac.jp (S.H.); f.w.ito@med.nagoya-u.ac.jp (F.I.); jiangli@med.nagoya-u.ac.jp (L.J.); yanatori@med.nagoya-u.ac.jp (I.Y.); samasuya@med.nagoya-u.ac.jp (Y.O.); akatsuka@med.nagoya-u.ac.jp (S.A.); 2 Center for Low Temperature Plasma Sciences, Nagoya University, Furo-cho, Chikusa, Nagoya 484-8601, Japan; 3 Sydney Medical School, The University of Sydney, Camperdown 2006, NSW, Australia

**Keywords:** iron, oxidative stress, ferroptosis, carcinogenesis, nanomaterial

## Abstract

**Simple Summary:**

Cancer is a major cause of human mortality worldwide. No life on earth can live without iron. Persistent oxidative stress resulting from continuous use of iron and oxygen may be a fundamental cause of carcinogenesis. Many animal models demonstrated that excess iron may lead to carcinogenesis. This is supported by a variety of human epidemiological data on cancer risk and prognosis. Cancer is basically a disease of the genome with persistently activated oncogenes and inactivated tumor suppressor genes through which iron addiction with ferroptosis-resistance is established. We predict that fine use of nanomaterials and non-thermal plasma may be able to reverse this situation.

**Abstract:**

Evolution from the first life on earth to humans took ~3.8 billion years. During the time there have been countless struggles among the species. Mycobacterium tuberculosis was the last major uncontrollable species against the human public health worldwide. After the victory with antibiotics, cancer has become the leading cause of death since 1981 in Japan. Considering that life inevitably depends on ceaseless electron transfers through iron and oxygen, we believe that carcinogenesis is intrinsically unavoidable side effects of using iron and oxygen. Many animal models unequivocally revealed that excess iron is a risk for carcinogenesis. This is supported by a variety of human epidemiological data on cancer risk and prognosis. Cancer is basically a disease of the genome with persistently activated oncogenes and inactivated tumor suppressor genes through which iron addiction with ferroptosis-resistance is maintained. Engineering has made a great advance in the past 50 years. In particular, nanotechnology is distinct in that the size of the engineered molecules is similar to that of our biomolecules. While some nano-molecules are found carcinogenic, there are principles to avoid such carcinogenicity with a smart possibility to use nano-molecules to specifically kill cancer cells. Non-thermal plasma is another modality to fight against cancer.

## 1. Introduction

Space started to expand through the Big Bang 13.8 billion years ago (Gya) [1] and Earth came into existence 4.6 Gya [2]. Evolution from the first life on Earth to humans took ~3.8 Gy [3]. At present, there are 1.75 million species on Earth [4]. Symbiosis of all the species on Earth is generally established in equilibrium currently, except for a fraction of endangered or extinct species, such as dinosaurs [5]. Some species are parasites of the other higher species [6]. There have been countless intense struggles among the species, which humans may sometimes call infection. The last major fight in the history of human public health was the one against mycobacterium tuberculosis, which was finally stopped by the discovery of antibiotics, such as streptomycin and isoniazid in the 1940s and 1950s [7].

Thereafter, in Japan, cancer has been the leading cause of death since 1981 (https://www.mhlw.go.jp/toukei/saikin/hw/jinkou/geppo/nengai19/dl/gaikyouR1.pdf), and mortality is still increasing. In the United States, some cancer mortality, such as colorectal cancer, is decreasing [8] presumably due to the success for early detection (secondary prevention) through screening with endoscopy [9,10]. However, nearly one third of the population dies from cancer in high-income countries worldwide (https://www.who.int/news-room/fact-sheets/detail/the-top-10-causes-of-death). The other major cause of death is atherosclerosis, leading to ischemic heart disease and stroke over time. However, we should not forget that the major causes of death in low-income countries are still various infections. Both cancer incidence and progression of atherosclerosis are proportionally age-dependent. In this review article, we consider the molecular cause of cancer from the highest global point of view, and then discuss the biological significance of nanomaterials and finally provide a perspective on the novel procedures to counteract cancer.

## 2. What Is the Major Cause of Carcinogenesis in Humans?

### 2.1. Epidemiology and Hypothesis

**Figure 1:**
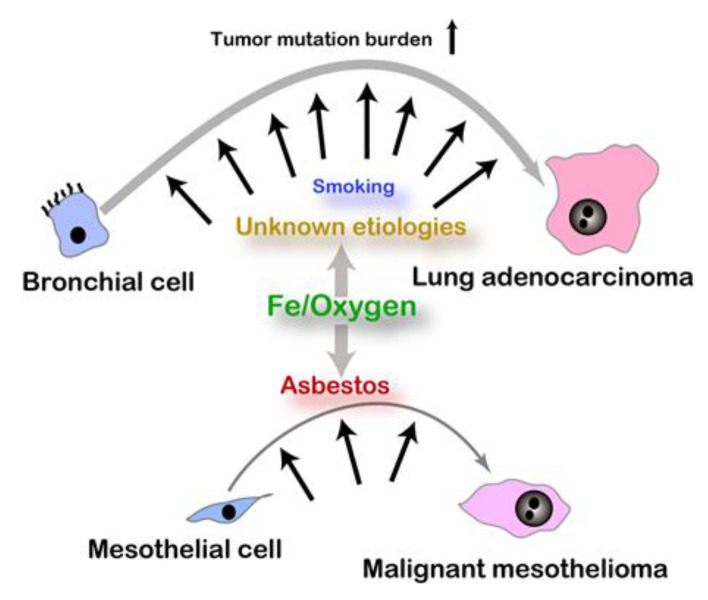
Etiology and somatic mutation burden in carcinogenesis. Asbestos exposure is a well-established risk for malignant mesothelioma whereas risks for lung adenocarcinoma with high somatic mutation burden are various and still vague.

After the end of longstanding countless wars against the other species, cancer is now one of the leading causes of human mortality in high-income countries worldwide. Here, it would be important to consider major causes of carcinogenesis in humans. We hypothesize that persistent use of iron and oxygen is the overlooked major cause of carcinogenesis in humans. Cancers may be classified into the two types: one with unequivocal risk factor(s), including endogenous and exogenous, and the other with more ambiguous or no identified risk factor(s), which is not necessarily consistent with a tumor mutation burden [11] (Figure 1). Typical examples of the first category are malignant mesothelioma (MM) by respiratory exposure to asbestos fibers [12,13] and hereditary breast/ovarian cancer in those with *BRCA1/2* mutations [14], namely occupational cancers and familial cancer syndromes. It is not surprising that these hereditary cancers share a relatively small fraction (5%~10%) even though there are hundreds of cancer-prone syndromes reported [15]. 

It is often difficult to identify the responsible risk(s) for most of the cancers, thus, falling into the second category. Current literatures discuss the importance of smoking [16] and a Western diet (high calorie, saturated oil, red meat, etc.) [17] as carcinogenic risks. We are not in a position to oppose these statements. Systematic reviews clearly reveal that smoking is a risk for various type of cancers including oral, laryngeal, lung, stomach, renal, and bladder cancers [18]. However, we believe that there are more fundamental factors to be considered as carcinogenic risks when looking at the steady global increase in the fraction of cancer as a major cause of human mortality where major infective diseases have been overcome. Furthermore, laboratory mice and rats suffer from a high incidence of cancer in old age [19,20,21] even if they are usually not exposed to apparent carcinogens or cancer-risk factors. Based on these facts, we started to consider the significance of the origin and evolution of life in carcinogenesis.

### 2.2. Iron, Sulfur, and Oxygen

As far as we are aware, no independent life on Earth can live without iron [22]. Geological studies revealed that the ancient sea contained a high concentration of catalytic ferrous iron [Fe(II)] when the first life on Earth was born [23,24]. It is true that iron is a fundamental element, existing in space because we can find many meteors which consist largely of iron [25]. Iron in the ancient ocean reacted with a subtle amount of oxygen to generate ores at the bottom of the sea [24]. Life might be defined as a continuous flow of electrons with reproductive activity of the next generation. Iron is a transition metal [26,27], efficient in electron flow, and, thus, preferentially used as a media for the first life on Earth [28]. 

Thereafter, a great oxidation event (GOE) occurred when an evolved life as cyanobacteria could transform light (solar) energy to electron flow to trigger rapid oxygenation [29]. Reportedly, sulfur was abundant in the environment at this period, where sulfur was firmly integrated in the life system not only as a coexistence of iron (Fe and S have a high affinity) but also as a competitor [24,30]. S is present in the sulfhydryl function of cysteine, which is in major use for reducing activity of polypeptides/proteins by counteracting as antioxidants. Representative ones are glutathione and thioredoxin, which are also used as a reducing unit for enzymes [31]. Furthermore, -SH works as an intracellular sensor for oxidative stress, as in the case of Keap1 and Nrf2 [32]. Recently, persulfides are regarded as a potent antioxidant mechanism [33].

After the GOE, the concentration of atmospheric oxygen started to rise gradually from ~0.6 Gya and reached a stable state of ~21% after several fluctuations [24]. Oxygen molecule, albeit a biradical, is relatively stable at the ground condition on the Earth and works flexibly as a media for electron flow. The most distinctive characteristic of O_2_ is its oxidizing ability, accepting a single electron to four electrons. At the same time, O_2_ is reduced ultimately to H_2_O. This process is quite versatile in that there can be electron flow of one to four, depending on the condition. O_2_ → O_2_^−^ (superoxide) → H_2_O_2_ → ^•^OH (hydroxyl radical) → H_2_O. In this case, the first two reactions are mediated by various enzymes whereas the third reaction is a chemical reaction designated as a Fenton reaction [27,34]. The hydroxyl radical is the most reactive species in the biological system on the Earth [35]. Therefore, higher animals hold and employ various enzymes, including catalase, peroxidases, and peroxiredoxins to directly decompose H_2_O_2_ to H_2_O [36,37,38]. In this way, the order of major elements used by the fundamental life is Fe → S → O during the evolution [39].

### 2.3. Excess Iron and Carcinogenesis

Excess iron is a soil for carcinogenesis [40,41,42]. Even though iron is essential for every kind of life on Earth, iron presents a double-edged sword. On the one hand, iron deficiency causes anemia (decrease in hemoglobin in the blood) and muscle weakness in higher animals [43]. On the other hand, iron excess causes oxidative damage to various different kinds of cells, which may lead to carcinogenesis [44,45,46]. Therefore, we have to consider both sides of the thresholds. Children and pregnant women definitely require a high amount of iron for the growth of organs. In the low-income countries, this issue is closely associated with malnutrition with food deficiency, but iron fortification to foods is recently recognized to alter gut microbiome [47]. Furthermore, we believe that supplementary iron intake to all the populations, irrespective of the iron status, whether deficient or sufficient, is not recommended [48,49]. This is partially because some form of iron, such as a nanoparticle form of iron, is absorbed from the duodenum without regulation via endocytosis but not via Fe(II) transporters [50].

Here, we briefly explain important principles on iron metabolism. More detailed descriptions are found in other recent publications [51,52]. Humans hold 2.5 to 4 g of iron in the body, which is the most abundant heavy metal, with zinc (2~3 g) [53] and copper (50~120 mg) [54] being the second and the third, respectively. In total, 60% of iron is in the heme of hemoglobin for oxygen transport (affinity of Fe[II] to O_2_) in red blood cells. Iron as a transition metal (Fe[II] ↔ Fe[III]) is important for DNA replication (ribonucleotide reductase), ATP synthesis (cytochrome oxidases), and antioxidant activity (catalase) in which either Fe(II) [55], heme [56], or the *Fe-S* cluster [57] is integrated as a cofactor of a catalytic subunit. Iron metabolism in humans as well as in other higher species is a semi-closed system, where only 1 mg of iron is absorbed from the villous surface membrane of duodenal epithelial cells and 1 mg is lost from the dead or peeled-off cells of skin or gastrointestinal system [41,58]. Therefore, iron from most of the dead cells inside our body is completely recovered by macrophages or their analogues or deposits in the interstitium.

**Figure 2:**
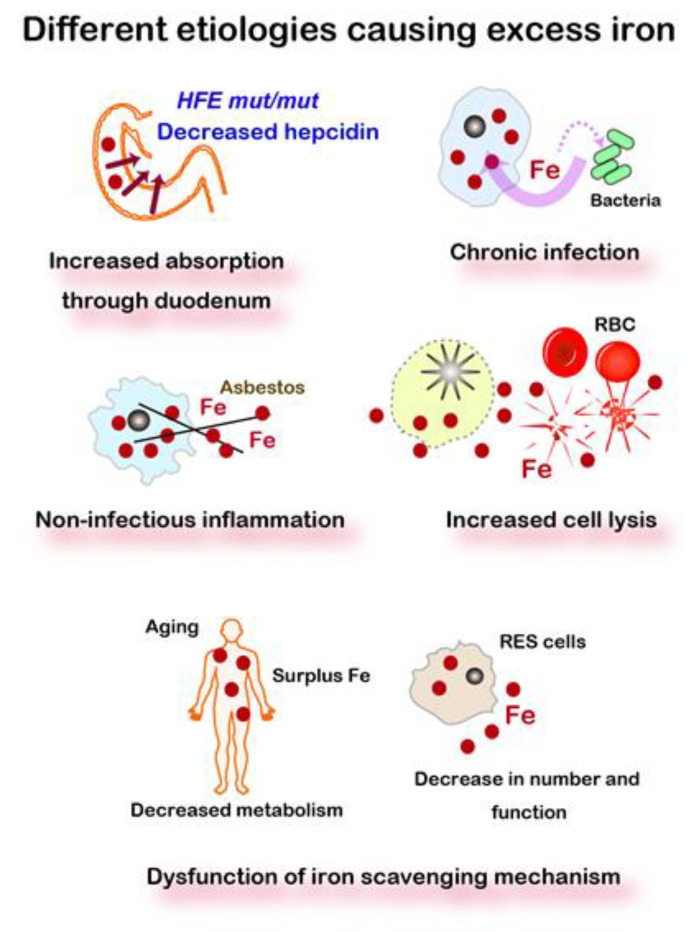
Five different etiologies causing excess iron. Iron metabolism in mammals is a semi-closed system, starting from the absorption at the duodenal epithelia but with no active excreting pathway. Various pathologies, including hemolysis, inflammation, and aging, lead to excess iron. *HFE*, a responsible gene for genetic hemochromatosis. RBC, red blood cells. RES, reticuloendothelial system, including macrophages, histiocytes, dendritic cells, Kupffer cells, and microglia. Refer to text for details.

Iron is essential not only for all the cells of the individual but also for the invading or coexisting lower species. Thus, every species competes for iron and fight with various smart molecular mechanisms, such as siderophores [59], for iron. As such, cells undertake to take up, reserve, and accumulate iron in themselves from dead cells or interstitium in higher species. Excess iron or iron overload often occurs in such pathologic conditions. Iron excess is classified into the following categories: (1) excess absorption via dysregulation (e.g., genetic hemochromatosis) or iron supplements, (2) chronic infection, (3) non-infectious inflammation (e.g., exposure to a large amount of foreign body difficult to be removed, such as asbestos), (4) increased cell death, including that of red blood cells (e.g., thalassemia, sickle cell disease), (5) relative decrease or dysfunction in an iron scavenging mechanism (e.g., aging), and (6) others, including repeated transfusion (Figure 2).

After briefly reviewing the molecular mechanisms associated with iron metabolisms, there are three independent lines of evidence available for the association of excess iron and carcinogenesis, (1) human observational data, either in specific diseases or in more broad population, (2) human interventional data, and (3) animal experiments. Representative human findings in the two categories are summarized in Table 1.

### 2.4. Iron-Induced Renal Carcinogenesis and Oxygenomics

Animal models are precious in that the comparisons among the experimental groups are the most precise due to the uniform genetic background (i.e., inbred strains) and living environment than the humans reported as epidemiological studies. There has been a key question whether Fe(II)-catalyzed the Fenton reaction of the repeated nature can induce carcinogenesis. The answer is positive. This came from a finding shed light by serendipity. Though iron is an important metal, the molecular understanding of iron metabolism required a long time and mostly started in the late 1990s. In the 1970s, only the transferrin system was recognized [51], but there was no in vivo method known to load iron to parenchymal cells of rodents. In those days, ferric nitrilotriacetate (Fe-NTA) was used to load iron to unsaturated transferrin in biochemical experiments [69]. NTA is a metal chelator with a structure of aminopolycarboxylic acid, solubilizing metals [70]. In the case of iron, both Fe(II)-NTA and Fe(III)-NTA are catalytic at neutral pH [26,71,72]. Intraperitoneal repeated injection of Fe-NTA to rats, for the first time, enabled iron loading to parenchymal cells (e.g., hepatocytes and β cells in Langerhans islets), showing similar signs of genetic hemochromatosis [73]. Unexpectedly, a long observation of this model revealed a high incidence (~90%) of renal cell carcinoma (RCC) with pulmonary metastasis in rats in 1982, and, later in mice, in the Department of Pathology, Kyoto University Faculty of Medicine [74,75,76,77]. 

At first, we could not imagine the responsible molecular mechanisms, but years later we found necrosis of renal proximal tubules with iron-catalyzed lipid peroxidation as early as 30 min after a single intraperitoneal injection of Fe-NTA [78,79,80]. Now, we sort out that this is ferroptosis *vide infra* [38,39,81], and this model unequivocally demonstrated that repeated oxidative stress catalyzed by iron leads to carcinogenesis in situ. This model contributed much to establishing oxidative stress markers, such as 4-hydroxy-2-nonenal (HNE) [82,83,84], 8-oxoguanine (8-oxoGua) [85,86], and thymine-tyrosine crosslinks [37,87]. 

**Figure 3:**
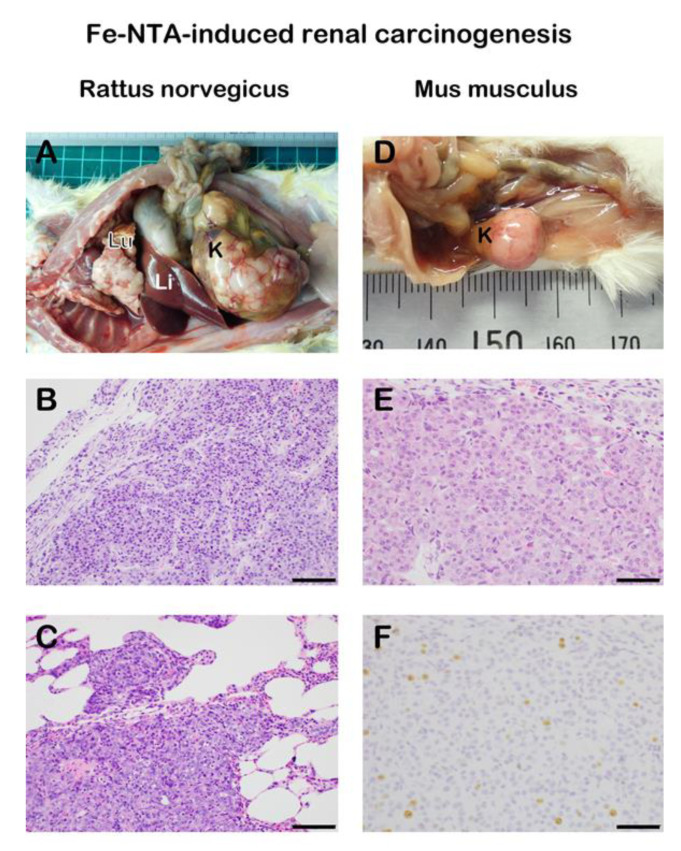
Species differences in ferric nitrilotriacetate (Fe-NTA)-induced renal carcinogenesis in *wild-type* rodents. Induction of an advanced renal cell carcinoma (RCC) with extensive pulmonary metastasis is frequently observed in rats whereas smaller RCC without metastasis is obtained with a much lower incidence in mice. (**A**) Macroscopic view of RCC with pulmonary metastasis and invasion in a male *wild-type Sprague-Dawley* rat 1 y after 11 weeks of repeated intraperitoneal 5–10 mg iron/kg Fe-NTA administration (3–5 times a week). Note primary RCC of 75 mm in diameter in the kidney and many metastatic nodules of 1–2 mm on the surface of lung. K, kidney. Li, liver. Lu, lung. (**B**) Histology of the primary RCC in the kidney. Proliferation of atypical glandular cells are observed in irregular glandular or solid structure (Hematoxylin and eosin staining). (**C**) Histology of the metastatic RCC in the lung. Similar adenocarcinoma to the primary site is invading the pulmonary alveolar structure. (**D**) Macroscopic view of RCC in a male *wild-type A/J* mouse 10 months after 12 weeks of repeated intraperitoneal 5–7 mg iron/kg Fe-NTA administration (6 times a week). Dose difference in the protocol between rats and mice comes from the difference in sensitivity to Fe-NTA. K, kidney. (**E**) Histology of the primary RCC in the kidney. Proliferation of atypical glandular cells are observed in irregular glandular or solid structure. (**F**) Ki-67 immunostaining of the RCC with an index of 5% (bar = 100 μm in B and C, 50 μm in E and F).

We later revealed that genetic alterations in this rat renal carcinogenesis are similar to those in human cancers in that the homozygous deletion of *p16^Ink4a^/p15^Ink4b^* tumor suppressor gene and amplification of *c-Met* oncogene are frequently observed [88,89]. Hemiallelic loss of the *p16^Ink4^* tumor suppressor gene is detected as early as three weeks after the start of Fe-NTA injections [90]. Furthermore, there are expressional and epigenetic alterations of substantial genes during carcinogenesis and tumor progression, such as annexin 2, thioredoxin-binding protein 2 (vitamin D_3_ up-regulated protein-1), and fibulin-5 [91,92,93,94,95]. Intriguingly, there is a marked difference between rats and mice regarding this renal carcinogenesis [96]. Most strains of rats (e.g., *Wistar, Fischer-344*, *Brown-Norway*, and *Sprague-Dawley*) provides a high incidence of renal cell carcinoma (RCC, 60–90%) whereas mice reveal a strain-specific susceptibility (e.g., *C57BL/6*, <10%, *A/Jackson*, ~60%). Grade of malignancy is also different. A half of RCCs metastasize to lung albeit *wild-type* animals in rats whereas a lower grade RCC is usually generated in mice with a low incidence of chromosomal aberrations [76,97] (Figure 3). Thus, our results on this RCC model confirm the fact that *Rattus norvegicus* are much closer than *Mus musculus* to *Homo sapience* in the evolutionary phylogeny.

This RCC model also opened an avenue to understand the site specificity of oxidative genomic DNA damage in the nucleus [98,99,100,101]. We have developed a technique, called DNA immunoprecipitation for oxidative DNA base modification (e.g., 8-oxoGua), and showed that distribution of oxidative DNA damage is not random but influenced not only by chemical species involved (e.g., ^•^OH and HNE) but also transcriptional activity, intranuclear localization (i.e., central or peripheral, near the nuclear membrane with Lamin B1 association) [102,103] and, thus, the structural fluctuation cycle [103,104]. We named such a research area as “Oxygenomics [98]”. Mutyh (an enzyme to repair 8-oxoGua in the genome)-deficient mice presented a higher incidence (26.7%) of RCC in comparison to *wild-type* mice (7.1%) [97].

### 2.5. Nanofiber-Induced Mesothelial Carcinogenesis and Excess Iron

Another important rodent carcinogenesis model associated with excess iron is asbestos-induced malignant mesothelioma (MM) [12]. This model also uses *wild-type* rats. Intraperitoneal injection of only 10 mg of asbestos (i.e., chrysotile, crocidolite or amosite, which correspond to white, blue, and brown asbestos, respectively) causes MM with an incidence of ~100% in two years [105]. Tremolite, which is a minor asbestos, also induces MM in rats [106]. This is extremely fast in comparison to human cases where 30~40 years of the latent period is usually after exposure to asbestos [66]. We believe that it is responsible that asbestos is directly exposed to mesothelial cells in our model whereas asbestos should go through lung parenchyma and pierce the visceral mesothelium to reach the parietal mesothelial cells in humans [13]. The essence of this carcinogenesis is local iron excess due to the affinity of asbestos to hemoglobin and phagocytic character of mesothelial cells [13,107,108]. Asbestos is a foreign material to our body, which is scavenged in situ through brave macrophages with resultant massive iron accumulation, which may be at least partially responsible for the deletion of the *p16^Ink4^* tumor suppressor gene [109]. Iron deposition is responsible not only from the adsorbed iron on the asbestos surface but also from the basic defense mechanism as inflammation to remove as much iron as possible in the competing extracellular environments to suppress virtual microorganisms [110]. We have shown preclinically that iron removal, either by redox-inactive iron chelators (deferasirox [111] and desferal [112]) or phlebotomy [113], is beneficial for the prevention of MM even after exposure to asbestos.

Carcinogenicity of asbestos depends not only on its physical dimension but also bio-durability as a fibrous mineral to reach pulmonary alveoli and further pleural cavity. Long (>20 μm) and thin (<250 nm) asbestos fibers can disrupt macrophages, which exacerbates inflammation and iron deposition [12,114]. *Mth1* (an enzyme to sanitize cytosolic nucleotide pool to remove 8-oxoGua) deficiency provided longer survival in asbestos-induced MM carcinogenesis, which meant that Mth1 is advantageous in crocidolite-induced mesothelial carcinogenesis in mice [115]. 

Of note, similar phenomena of the association between iron excess and carcinogenesis were reported on multiwalled carbon nanotubes (MWCNT) [116], which strictly depend on the diameter of the MWCNT [117,118]. MWCNT, which is a fibrous synthetic product purely from carbon, was discovered in 1991 [119], and is abundantly used to lengthen the lifetime of electric battery to strengthen rubber with thermal/electric conductivity and to compose biomedical sensors as hybrid composites with graphene [120]. MWCNT with a diameter of ~50 nm can cause MM when injected intraperitoneally [117,121]. Of note, homozygous deletion of *p16^Ink4a^/p15^Ink4b^* tumor suppressor gene is observed in almost all the cases of MM induced [117], which is the same for asbestos-induced MM. All of these results indicate that *p16^Ink4a^/p15^Ink4b^* tumor suppressor gene is a major target in excess iron-associated carcinogenesis [13,71,114,122]. Since the *p16^Ink4a^/p15^Ink4b^* tumor suppressor gene is the second major mutated gene in human cancers only after TP53 [123], we believe that persistent use of iron and oxygen is one of the major causes of human carcinogenesis.

### 2.6. Resistance to Ferroptosis

Light microscopy can differentiate apoptosis from necrosis morphologically. Apoptosis reveals nuclear and cytoplasmic fragmentation through caspase activation with little inflammatory responses [124] whereas necrosis generally shows cytoplasmic swelling with nuclear pyknosis and inflammatory responses. This is still a golden rule at present [125]. Formerly, necrosis was defined as an uncontrollable nature of passive cell death due to high levels of injury. Now, the concept of regulated necrosis is established, where some form of necrosis requires signal activation (i.e., not passive) and takes some time (i.e., mins to hours) for its execution [39]. 

**Figure 4:**
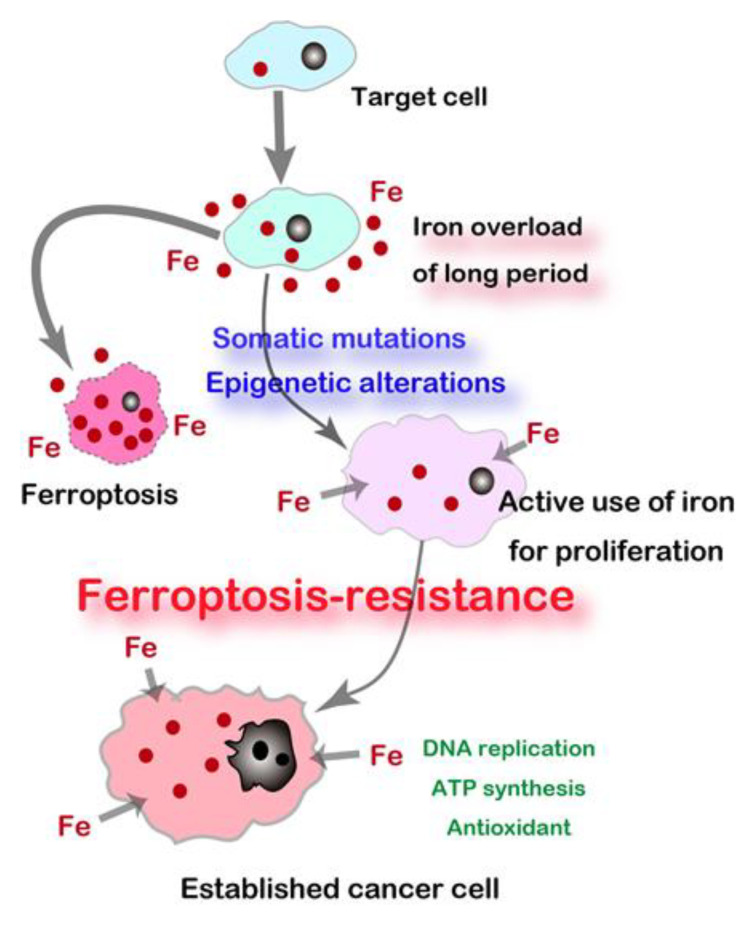
Carcinogenesis as a process to establish “Iron addiction and ferroptosis-resistance”. Long-term iron overload is a soil for carcinogenesis.

Regulated cell death is currently divided into 12 different forms [125], among which ferroptosis was coined in 2012 [126]. Ferro- indicates Fe(II) whereas -ptosis means falling off. Ferroptosis is defined as a catalytic Fe(II)-dependent regulated necrosis accompanying lipid peroxidation [81]. Ferroptosis was first reported on the treatment of erastin (i.e., an inhibitor of cystine/glutamate antiporter, SLC7A11) on *N-Ras* mutant fibrosarcoma cells during the drug screening for *Ras*-activated cancers [126]. We immediately noticed that renal tubular necrosis induced by Fe-NTA [78,80,82] as described above is ferroptosis [38]. As an intriguing coincidence, ferroptosis of renal proximal tubules occurs after conditional knockout of glutathione peroxidase 4 (GPX4), which is the only membrane-specific isozyme of glutathione peroxidase [127]. Currently, we interpret that this is a fight between Fe and S and that a significantly higher Fe/S ratio than the control leads to ferroptosis [39]. Cancer cells require a high amount of iron to replicate DNA, proliferate, and invade. Therefore, they are rich in catalytic Fe(II) [128,129]. Carcinogenesis is a process to obtain this resistance to ferroptosis as shown in rodent RCC and MM models [30] (Figure 4).

### 2.7. Cancer Prognosis and Iron Metabolism

We have, thus, far discussed the iron-induced carcinogenic mechanisms. Here, we would mention the effects of iron deficiency or excess on the prognosis of cancer in humans. Several national surveys were performed in the 1980s and 1990s in the US and Finland. For example, 3287 men and 5269 women participated in the first national nutritional survey in which men and women were divided into five groups, based on baseline transferrin saturation (<30%, 30–40%, 40–50%, 50–60%, 60%<). For men and women combined, cancer risk for each group relative to the first was 1.0, 0.95, 1.16, 1.38, and 1.18 whereas mortality for each group was 1.0, 0.96, 1.22, 1.29, and 1.73 [130]. Other studies are summarized in a previous review article [40]. Here, we summarized the recent representative data in Table 2. Most of the data suggests that iron-rich status provides poorer prognosis in cancer patients.

## 3. Association of Cutting-Edge Engineering and Cancer

### 3.1. Nanomaterials and Carcinogenesis

Nanomaterials are defined as a material that contains at least 50% of the particles (by number) in the 1–100 nm range [135]. These materials are novel in that the dimension of the molecules generated through new developments are as small as the levels of our own biomolecules persistently used in our daily metabolism. It was socially meaningful to find that some of the fibrous nanomaterials (i.e., multiwalled carbon nanotube [MWCNT] of 50 nm diameter) are carcinogenic in rodents, causing MM after intraperitoneal administration (*vide supra*) [117].International Agency for Research on Cancer, thereafter, designated MWCNT of 50-nm diameter as Group 2B (possible human carcinogen) and other MWCNTs as Group 3 [136]. It was later reported that inhalation of MWCNT of a 50-nm diameter causes lung carcinoma in rats [137].

This kind of information is precious to differentiate management of MWCNT of different diameters toward safer work environments. MWCNT is already providing us with daily convenience as a high-power battery for smart phones and highly durable rubber for car tires and excavators [120]. The robotics automation process in the factory and avoidance of carcinogenic MWCNTs are helpful to decrease the carcinogenic risks for humans. We demonstrated in a 3-year rat study that MWCNT of 15 nm (tangled form) is not carcinogenic by intraperitoneal injection [121]. Based on these results, we believe that a subacute study by intraperitoneal injection with a four week observation predicts the carcinogenicity of the bio-persistent fibrous material, such as asbestos and MWCNT [117,118]. Commercial chlorine bleach can degrade MWCNT to CO_2_ ex vivo, which would facilitate the disposal of this nanomaterial [138].

Furthermore, it was recently reported that WS_2_ and MoS_2_ nanosheets (two-dimensional transition metal dicharcogenides [139]) induces ferroptosis through surface vacancies in bronchial epithelial and macrophage cells [140]. This is also an airborne risk and can be prevented by prior methanol treatment to passivate active particle surfaces [139].

### 3.2. Nanomaterials for Cancer Treatment by Designing the Death Code

**Figure 5:**
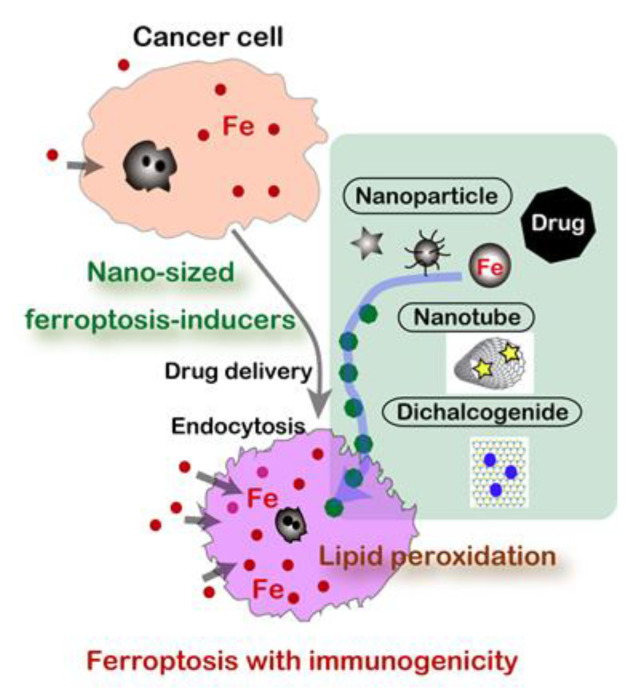
Nanomaterials as novel ferroptosis inducers for cancer cells.

Conversely, nanomaterials may be able to specifically kill cancer cells if designed optimally by exploring a structure-activity relationship (Figure 5). In this century, nanomaterials have been recognized as emerging media for drug delivery and a number of clinical trials are in progress. Currently, regulated cell death is classified into twelve and each has a fixed death code [125]. There is a huge possibility that nanomaterials can initiate and modify death codes in which ferroptosis acquired a high attention [141].

When we understand that carcinogenesis a process to establish “iron addiction with ferroptosis resistance” [13,30], cancer cells are expectedly rich in catalytic Fe(II) [128,129] in the cytosol to be easily utilized for enzymes toward unregulated endless proliferation, such as ribonucleotide reductase (DNA synthesis), cytochrome oxidase (ATP synthesis), and catalase (antioxidant). Fe(II) [55], *Fe-S* cluster [57], and heme [56] are important cofactors for these enzymes. Thus, this is the strategy to induce ferroptosis by using nanomaterials specifically in cancer cells, but not in non-tumorous cells. Most of the nanomaterials are actively taken up by cancer cells through endocytosis. This is an active research area in material science, and various forms of iron-based nanomaterials are preclinically proposed, including iron oxide nanoparticles (IONs) [142], lipid-hydroperoxide-tethered IONs [143], assembled IONs [144], amorphous iron nanoparticles [145], iron-organic frameworks [146], and FePt nanoparticles [147] in addition to small molecule chelators [148]. 

### 3.3. Non-Thermal Plasma

Plasma is the fourth condition of a physical state, which presents the highest energy over gas with ionization [149]. High-temperature plasma has been used from the 1960s for manufacturing semiconductors. Development of modern electronics produced plasma of a near body temperature (i.e., non-thermal plasma (NTP) or low-temperature plasma) [150,151,152]. Inert gas, such as Ar or He, is used as flow supply with high voltage/electron density to generate various reactive species from atmospheric O_2_ and N_2_, including ^•^OH, H_2_O_2_, O_2_^−^, and NO. Fine adjustment of the concentration of each gas and humidity provides different fractions of each reactive species [150,153].

**Figure 6:**
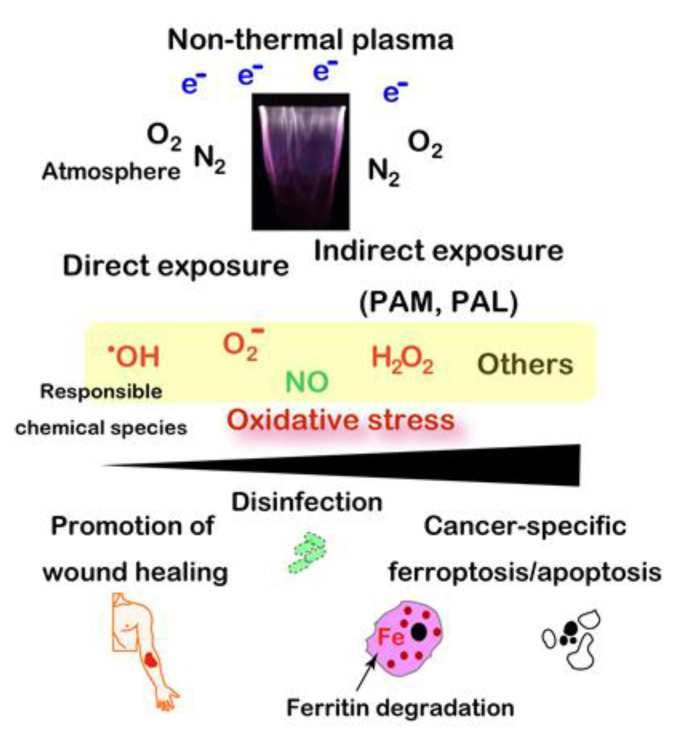
Wide applicability of non-thermal plasma to biomedical field, including specific ferroptosis inducer for cancer cells. PAM, plasma-activated medium. PAL, plasma-activated Ringer’s lactate solution.

NTP was established as a novel method to load oxidative stress to the target coordinates [154]. In addition to direct exposure of NTP, plasma activated media and lactate (PAM [155,156,157,158] and PAL [159], respectively) are under intensive investigation even though the responsible chemical species have not been completely identified at present. NTP as preclinical experiments can be applied to multiple medical and biological purposes [152], including: (1) disinfection of viruses and bacteria, (2) promotion of wound healing, (3) specific killing of cancer cells [151,160,161], (4) removal of endometriotic lesions [162,163], and (5) increasing yield of plants [164] and fish [165]. The final biological effects depend on the relative strength of the oxidative stress loaded [37] (Figure 6).

Regarding the specific killing of cancer cells, abundance of catalytic Fe(II) in cancer is important for the effects of NTP to cause the Fenton reaction eventually to ferroptosis [160,166]. Here, NTP-induced ferritin degradation with a simultaneous reduction to Fe(II) may be important [167]. This strategy is to attack the Achilles’ heel of cancer cells [39], which they obtained for their fundamental existence through the evolutionary process as discussed *ibid*. The drawback of NTP is that it reaches only a few mm in depth [154]. Thus, it would work for surface tumors in the situations of somatic cavity (e.g., peritonitis carcinomatosa) or operational margins, where other modalities are not presently easily applied.

## 4. Conclusions

Animal models suggest that carcinogenesis can be a side effect of using iron and oxygen for decades whereas there is a long list of carcinogenic agents. We may interpret that carcinogenic agents in the lists are intensifying the side effects of iron and oxygen. Animal models contributed to establish the concept of carcinogenesis as “iron addiction with ferroptosis-resistance”. Alternatively, there is a huge possibility to specifically kill cancer cells by attacking this Achilles’ heel of cancer cells with ultimate bioengineering.

## Figures and Tables

**Table 1:** Representative human facts on the association of iron and carcinogenesis.

Observational Findings			
Specific Diseases	Cancer	Etiology of Excess Iron	References
Genetic hemochromatosis	Hepatocellular carcinoma, etc.	iron sensor dysfunction	[60]
β-Thalassemia	Hepatocellular carcinoma	transfusion and HBV/HCV	[61]
Viral hepatitis C	Hepatocellular carcinoma	low hepcidin	[62,63]
Ovarian endometriosis	Clear cell carcinoma, etc.	monthly hemorrhage	[64,65]
Asbestos exposure	Malignant mesothelioma	foreign body, adsorption	[12,13,66]
**Biomarkers of Iron Stores**			
Transferrin saturation	Non-skin cancer	HR = 1.68, 95% CI = 1.18 to 2.38, *p* < 0.01	[67]
**Interventional Study**			
Phlebotomy (500 mL× 2 × 4.5 y)	Visceral malignancy	HR = 0.65, 95% CI = 0.43 to 0.97, *p* = 0.036	[68]

HBV, hepatitis B virus. HCV, hepatitis C virus. HR, hazard ratio. CI, confidence interval.

**Table 2:** Representative human facts on the association of iron and cancer prognosis.

Biomarker for Poor Survival	Cancer	Facts	Reference
Serum ferritin (≥150 ng/mL)	mCRC	HR = 1.68, 95% CI = 1.18 to 2.38, *p* = 0.007	[131]
*ibid.*	advanced NSCLC	HR = 1.81, 95% CI = 1.24 to 2.64, *p* = 0.002	[132]
*ibid.* (≥267 ng/mL)	HCC after hepatectomy	HR = 1.651, 95% CI = 1.213 to 2.247, *p* = 0.001	[133]
Transferrin receptor (CD71)	Breast cancer	independent prognostic marker in ER+ cohort	[134]

mCRC, metastatic colorectal cancer. NSCLC, non-small cell lung cancer. HCC, hepatocellular carcinoma. ER, estrogen receptor.

## Abbreviations

Fe-NTA	ferric nitrilotriacetate
GOE	great oxidation event
GPX4	glutathione peroxidase 4
Gya	billion years ago
HNE	4-hydroxy-2-nonenal
ION	iron oxide nanoparticle
MM	malignant mesothelioma
MWCNT	multiwalled carbon nanotube
NTA	nitrilotriacetate
NTP	non-thermal plasma
8-oxoGua	8-oxoguanine
RCC	renal cell carcinoma
SWCNT	single walled carbon nanotube
